# ZD6474 reverses multidrug resistance by directly inhibiting the function of P-glycoprotein

**DOI:** 10.1038/sj.bjc.6604077

**Published:** 2007-11-06

**Authors:** R I Freshney

**Affiliations:** 1 24, Greenwood Drive Berasden, Glasgow G61 1BD, UK

**Sir**,

Although the role of this compound in multidrug resistance may well be important and the observations valid, the two cell lines that they have used are not as described in their article. MCF-7-ADR is not a drug-resistant variant of the MCF-7 breast carcinoma cell line but is, in fact OVCAR-8 an ovarian carcinoma cell line (Liscovitch M, Ravid D (2007). *Cancer Lett* **245**(1–2): 350–352) and KB cells are not oral carcinoma but were cross-contaminated with HeLa many years ago (European Collection of Cell Cultures (2002). HeLa contamination – an old problem that has not gone away! ECACC Newsletter, March 2002; Stacey GN, Masters JRM, Hay RJ, Drexler HG, MacLeod RAF, Freshney RI (2000). Cell contamination leads to inaccurate data: we must take action now. *Nature* **403**: 456). Clearly, these authors (like many others!) are unaware of this.

I suggest that future instructions to reviewers contain advice on the scrutiny of cell lines used in such publications to ensure that similar errors do not occur in the future. It is a simple matter for a reviewer to access the website of a recognised cell bank, such as ECACC (www.ecacc.org.uk) or ATCC (www.atcc.org) where information can be obtained as to the authenticity of cell lines in common use. Authors should also be asked to provide hard evidence of the authenticity of the cell lines that they use as a condition for accepting a manuscript for review.

